# Small-Scale and Occluded Pedestrian Detection Using Multi Mapping Feature Extraction Function and Modified Soft-NMS

**DOI:** 10.1155/2022/9325803

**Published:** 2022-10-11

**Authors:** Addis Abebe Assefa, Wenhong Tian, Kingsley Nketia Acheampong, Muhammad Umar Aftab, Muhammad Ahmad

**Affiliations:** ^1^School of Information and Software Engineering, University of Electronic Science and Technology of China, Chengdu, China; ^2^Department of Computer Science, National University of Computer and Emerging Sciences, Islamabad, Chiniot-Faisalabad Campus, Chiniot 35400, Pakistan

## Abstract

In autonomous driving and Intelligent transportation systems, pedestrian detection is vital in reducing traffic accidents. However, detecting small-scale and occluded pedestrians is challenging due to the ineffective utilization of the low-feature content of small-scale objects. The main reasons behind this are the stochastic nature of weight initialization and the greedy nature of nonmaximum suppression. To overcome the aforesaid issues, this work proposes a multifocus feature extractor module by fusing feature maps extracted from the Gaussian and Xavier mapping function to enhance the effective receptive field. We also employ a focused attention feature selection on a higher layer feature map of the single shot detector (SSD) region proposal module to blend with its low-layer feature to tackle the vanishing of the feature detail due to convolution and pooling operation. In addition, this work proposes a decaying nonmaximum suppression function considering score and Intersection Over Union (IOU) parameters to tackle high miss rates caused by greedy nonmaximum suppression used by SSD. Extensive experiments have been conducted on the Caltech pedestrian dataset with the original annotations and the improved annotations. Experimental results demonstrate the effectiveness of the proposed method, particularly for small and occluded pedestrians.

## 1. Introduction

Pedestrian detection is a fundamental task in computer vision applications such as surveillance, robotics, and automotive safety. Specifically, pedestrian detection in transportation is significant because it can save countless lives [1]. Despite extensive research on pedestrian detection, new studies show significant advances, signaling that a maximum threshold has yet to be reached, i.e., small-scale pedestrian detection and occlusion are two of the current state-of-the-art constraints. Insufficient feature strength in small objects and the stochastic nature of kernel initialization are the leading cause of incapability in detecting small objects [2, 3].

Occlusion is another challenging issue in pedestrian detection because it is difficult to compromise miss rate and accuracy when detectors are sensitive to the nonmaximum suppression (NMS) threshold in crowded environments [4]. The technology for detecting pedestrians is advancing all the time. Although the occlusion problem can be solved, there is still a significant barrier to overcome.

Videos taken by businesses such as banks and shopping malls obscure the majority of people on the street [5]. When pedestrians are hidden by background clutter or other objects, it can be more difficult to detect them. In the field of smart cities, pedestrian detection under occlusion has become a popular method of tracking people. Occupied pedestrian detection is useful in a wide range of fields, such as automated driving, intelligent video surveillance, robotics, human-computer interaction, and security. Assisted driving and self-driving vehicles are two of the most important aspects of intelligent transportation. It is essential to detect humans even when they are partially obscured by objects. Drivers need to be aware of pedestrians and give them the benefit of the doubt when it comes to pedestrian detection under occlusion.

There are four levels of occlusion between pedestrians [6]: zero, one to 35%, 35 to 80%, and above 80%. Depending on the detection framework, occupied pedestrian detection can be broken down into two approaches: (1) traditional methods [7, 8] and (2) deep learning methods [9–12].

Traditional methods for dealing with occluded pedestrians include combining hand-engineered kernel features such as the Histogram of Oriented Gradient (HOG) descriptor [8], Scale Invariant Feature Transform (SIFT) [13], and aggregated channel feature [14] with linear support vector machine [15] or random forests [16]. The ability of HOG and SIFT to represent distinguishing characteristics of a pedestrian makes them popular algorithms. Many pedestrian detection frameworks have been presented to address a wide range of detection challenges, from the occluded to the visible and small to large scale.

Dollar et al. [17] presented Integral Channel Feature (ICF), which uses integral images to extract features from HOG and LUV color channels (HOG + LUV) and employs boosted decision forests for pedestrian detection to boost detection accuracy. Moreover, Dollar et al. developed three opposing cascaded modules (soft cascade, excitatory cascade, and inhibitory cascade) to maximize the inference rate. After ICF, handcrafted features with improved LUV color channels (Aggregated Channel Features (ACF), Rotated Filters [18], Locally Decorrelated Channel Features (LDCF) [19], Checkerboards [20], and SquaresChntrs [21]) were used to improve low-resolution image detection and enable detector invariance to changes in lighting conditions.

The alternative approach to tackle occluded pedestrians is the component-based method. Even if a portion of the pedestrian to be detected is obscured, the remaining parts can be used to determine the pedestrian's location. According to Leibe et al. [22], pedestrian detection algorithms in crowded scenes are equivalent to the prototype of pedestrian detection under occlusion. A key component of their approach is the use of probabilistic top-down segmentation to combine local and global cues. To better deal with pedestrians, Mohan et al. [23] found that pedestrians can be divided into four sections: head and shoulder, leg, left hand, and right hand. It is more effective to deal with occlusion. Occlusion-aware pedestrian detection frameworks were devised using a deformable part-based model (DPM) [24], which generalizes global appearance from local appearance. The first method produces acceptable detection results with minimal computational effort. Despite this, it has a high miss rate, has difficulty determining the proper aspect ratio between image pyramids and sliding window step size, and kernels are not learnable, meaning they must be constructed manually.

The second approach is based on deep learning and significantly boosts detection accuracy and response time. The approach to deep learning is primarily divided into two categories. First, there is the two-stage detector algorithm, which separates target recognition and location into two parts. For example, the family of recurrent convolutional neural networks (R-CNNs) has been used in [25, 26] for object detection. R-CNN is one of the most prominent deep learning-based object detection frameworks. Since then, the R-CNN family has been subjected to various studies to increase detection accuracy and response time. A super-resolution algorithm, a combination of handcrafted features with convolutional neural network (CNN) feature maps [11, 27–31] and CNN's layer fusion are the leading approaches built on top of the R-CNN family to deal with low-resolution, occlusion, and normal (fully visible and large scale) pedestrian detection.

Tian et al. [32] proposed the Deep-Parts, using Deep Part, they can automatically select critical body parts for occlusion handling from a part pool that includes parts of all sizes. An ensemble of detectors is learned and the output of the ensemble is integrated as a strategy for dealing with occlusion in these methods. However, the procedure is difficult and extensive. Furthermore, by combining a faster R-CNN with an attention mechanism composition [33], this method has a minimal training burden, making it easy to train. The overhead for this approach is minimal, making it simple to train. The use of attention mechanisms in CNN object detection has been widespread. In order for the detector to focus more on visible body parts, an additional mechanism has been added. The primary method for dealing with small-scale or low-resolution pedestrian detection is to increase the feature's resolution.

Tesema et al. [34] blends handcrafted features with convolutional feature maps to detect a low-resolution pedestrian. Chu et al. [35] synthesized high-level features using features from all levels. By adaptively merging multilayer features, Liu et al. [36] suggested a gated feature extraction module. Wang et al. [37] proposed a multiscale area proposal network with a decision forest for categorization to deal with scale-invariance differences. Inspired by the human detection and recognition mechanism, attention models have been developed and integrated on R–CNN for different tasks. For example, Zhang et al. [38] observed that individual streams depict different regions of an object and employed channel-wise attention [39] to detect occluded pedestrians. The maximum detection accuracy is achieved by all pedestrian detection frameworks built on top of the R-CNN family. However, they are complicated, with many moving parts, and can only be used for offline deep learning-based object detection.

Second (class-aware), it is a one-stage detector algorithm that includes the single shot detector (SSD) [40] and You Only Look Once (YOLO) [12, 41] object detection framework has been developed to address these concerns. The SSD object detector is entirely end-to-end, has no complex moving parts, and can perform in real time. The current state-of-the-arts built on top of class-aware frameworks yields a good result in the best conditions, such as a pedestrian with a comparable scale (above 80 pixels height) and complete visibility. However, detection and recognition capabilities for small-scale pedestrian and partial occlusion are severely compelled.

The poor performance of the state-of-the-art works in detecting small-scale or obstructed pedestrians is primarily due to the stochastic nature of kernel initialization, fading of an object resolution as feature maps traverse through deep layers, and the greedy nature of the SSD's nonmaximum suppression approach. The SSD begins with a base network (VGG-16). Later, convolution and pooling layers are replaced with a set of new convolution layers. The output fully connected (FC) layer is connected to each CONV layer. The most common complaint of SSDs is that they do not function well for small items because small objects do not always exist on all feature maps. Hence, the curse of this approach for small-scale pedestrian detection is that there is a high probability of losing all of the features of an object, i.e., reduction of resolution.

In other words, SSDs do not function well for small items because small objects' attributes do not always exist on all layers of the region proposal module. Besides, occlusion creates a challenge in detecting pedestrians, especially in a crowded situation. In the Caltech pedestrian dataset [5], for example, notes that other pedestrians occlude 70 percent of pedestrians. It is challenging to compromise miss rate and accuracy when detectors are sensitive to the threshold of nonmaximum suppression (NMS) in crowded settings.

**Figure 1:**
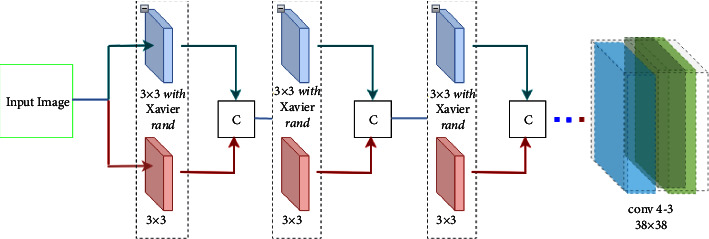
Our proposed backbone module. It has two branches i.e., the bottom branch is a sample layer of the VGG-16, and the top component is a new integrated layer in which the kernel size is equal in size to the bottom one except for weight initialization which is Xavier. At the end of each layer, feature maps are extracted from both branches, and combined **C** in-depth wise.

We propose a solution to overcome the challenges mentioned above; we offer a new architecture with a channel-aware attention feature fusion and nonmaximum suppression that considers score and IOU parameters to reduce a miss rate and increase accuracy. Our architecture adopts VGG-16 [42] by adding a new branch with the same kernel size as the original architecture. However, we employ Xavier [43] weight initialization, which diffuses the concentration in the center out to the periphery, unlike normal Gaussian distribution [44] used by VGG-16 weight initialization. So, a combination of features from both branches can magnify the effective receptive field. We also propose a channel-aware attention feature fusion between each CONV layer of the region proposal module, as shown in Figure 1, to overcome the vanishing of features on a small scale. In addition, to handle pedestrians with occlusion, we propose nonmaximum suppression which is a modified version of Soft-NMS. Specifically, we consider the difference between the objects proposals score parameter and an intersection (IOU) used by soft-NMS to reduce the miss rate caused by the greedy nature of nonmaximum suppression. The main contributions made in this work are as follows:

1. We propose a framework that incorporates channel-aware feature fusion with a heterogeneous mapping function to efficiently employ all features in a receptive field while avoiding vanishing gradients generated by a small-scale object.
2. We explore how the field of view of a unit value in a particular layer in the network is affected by the kernels' weight initialization. Our experiments show that Gaussian random weight initialization assigns a large scale at the center of the kernel and a small value for the outermost; as a result, only the central receptive field has a high impact on unit out. We find that equal levels of the relevance of features within the receptive field enable a framework to attain maximum effective receptive field without a shallow layer.
3. We find that applying greedy nonmaximum suppression to crowded pedestrian detection reduces precision by excluding detection with high scores that are likely to be false positives. To overcome these limitations, we employ an adaptive bounding box suppression.
4. Experiments and analyses are conducted on the Caltech pedestrian dataset [5] with the original and new annotations. Our approach achieves the miss-rate (MR) of 9.1 and 6.08, outperforming detection with occlusion performance.

The remainder of this article is organized as follows: Section 2 goes through the related work.  Section 3 goes through the proposed architecture and its components in detail. In Section 4, we conduct experiments, analyze the result, and conduct an ablation study. Finally, we sum up our work and make some recommendations for further research.

## 2. Related Works

So far, pedestrian detection studies have yielded positive results in ideal conditions such as large scale, full visibility, and high resolution. Despite its great success, pedestrian detection still faces a number of challenges under certain conditions, as discussed below.

### 2.1. Scale Aware Pedestrian Detection

The current state-of-the-art in small-scale pedestrian detection methods focuses on enhancing weak signals in small-scale objects and preserving that signal while applying feature extraction operations such as convolution and pooling. Hybrid channel fusion and pure CNN feature maps are the two main approaches to small-scale pedestrian detection. They merged the advantages of handmade and CNN kernels in hybrid approaches, integrating high-resolution, and easy-to-compute handcrafted feature channels with low resolution and computationally expensive CNN feature channels.

On the other hand, some approaches use the handcrafted feature for region proposals. CNN for categorization applies CNN for bounding boxes generation while based on the extracted feature shallow classifier. The handcrafted filtered channel features (FCF) [20] were proposed to be replaced by convolutional channel features (CCF), in which each pixel in the final convolutional layer is treated as a single feature by Yan et al. [45].

Hu et al. [46] trained an ensemble of boosted decision forests using features from different layers of a CNN. Zhang et al. [47] and Tesemaa et al. [34] used the region proposal network (RPN) as an initial pedestrian detector, then trained a shallow classifier with in-depth features to refine detection results. Li et al. [48] proposed extracting multi-resolution in-depth features from different convolutional networks. Sheng et al. [48] created a filtered channel framework that combined deep semantic segmentation features with shallow handcrafted channel features. Wang et al. [37] created a multi-scale region proposal network that included a decision forest for classification to deal with scale differences.

The other approach is detecting objects on multiple scales [21] suggests a cohesive multiscale CNN (MS-CNN) that detects objects at multiple intermediate layers and up samples to avert feature map resolution issues when dealing with small instances. Instead of a single downstream classifier, the fused deep neural network (F-DNN + SS) method [49] employs a derivation of the faster R-CNN framework fusing multiple parallel classifiers using soft-rejection, such as Resnet and Google net, as well as pixel-wise semantic segmentation in postprocessing to suppress background proposals. The other problem is related to pose and learning method, for example, Wiedemer et al. and Tang et al. [50, 51] demonstrate that the combination of supervised domain adaptation with fine-tuning is effective in tackling settings posses caused by geometric distortions, perspective changes, and the scarcity of training samples. This paper suggests a multifocus feature extractor base module that combines feature maps extracted from the Gaussian and Xavier mapping functions in order to improve the effective receptive field which results in a signal of small-scale object to be used effectively.

### 2.2. Occlusion Aware Pedestrian Detection

The occlusion of pedestrians is a typical problem. For instance, 53% of pedestrians are obscured in the Caltech pedestrian dataset. The two types of pedestrian occlusions are interclass and intraclass occlusions. When other objects obstruct pedestrians, this is known as interclass occlusion (not pedestrians). Intraclass occlusion occurs when other pedestrians obstruct pedestrians. To handle interclass occlusion, emphasize the unoccluded part's characteristics while repressing the occluded part's attributes. To cover occlusion patterns [32, 52, 53], for example, learn different parts of body detectors. The challenging part of this multipart detector is combining the results. For the proposed model, taking into account the aspect ratio of a human's height and width, we structure a region proposal scheme.

The algorithm generates multiple bounding boxes with varying scales and aspect ratios, each centered on a single pixel. This configuration of human-based anchors enables the model to perform exceptionally well when determining whether a particular type of pedestrian appears in an image. We also include a small aspect ratio with equal height and width to enable the model to detect a pedestrian that is visible only above the neck. In addition, in the Caltech pedestrian dataset [5], the occlusion statistics indicate the number of occlusions caused by a pedestrian (intraclass). Therefore, the preceding setup enables the proposed model to predict a higher detection precision for intraclass occlusion.

Annotating the visible part of a body [54–59] is the other method; this technique consumes resources for annotation. NMS must remove duplicate bounding boxes when detecting pedestrians. As an adverse effect, NMS merges the bounding boxes of different pedestrians in a crowd scene (intra-class occlusion). Improved NMS strategies, such as dynamic NMS threshold, are one solution to this problem. To combat high miss rates caused by greedy nonmaximum suppression, we propose an adaptive bounding box suppression that takes score and Intersection Over Union (IOU) parameters into account.

## 3. The Proposed Architecture

This section introduces our proposed architecture, its components, a few fundamental design principles, and experiments and analysis. The proposed framework incorporates three submodules: the backbone module, the dense multiscale region proposal module, and the adaptive bounding box suppression module. The complete pipeline is presented in Algorithm 1.

### 3.1. The Backbone Module

The backbone-network extracts salient features from the input image. Using the pretrained ImageNet [60] model as the basis network offers multiple benefits. The benefit includes the flexibility to incorporate several cutting-edge models, the elimination of the need for a powerful machine to train, and a faster training period. However, the ImageNet dataset contains objects of “medium” size, spanning from 60 to 140 pixels, outside the intended domain, such as when a dataset is in small scale or occlusion. In addition, A single-scale kernel feature extractor like VGG16 has few parameters compared with a multiscale feature, but the signal strength at the end of a layer is low when compared with a multiscale feature.

As a result, we propose a new architecture that adopts VGG-16 by appending a new branch layer with the same kernel size as the original architecture. However, we apply Xavier [43] weight initialization, which diffuses the concentration in the center out to the periphery, unlike normal Gaussian distribution [44] used by VGG-16 weight initialization. Our base network is a multi-feature extraction model like Google net [61], mobileNet [62], and ResNet [63]. They can capture salient features of an image, enabling us to get a maximum effective receptive field with a shallow layer and enabling the region proposal regression module to get a sufficient signal. However, they contain many parameters when compared with our proposed base network. Finally, we combine feature maps from an integrated branch layer and original feature maps into channel dimensions as shown in Figure 1.

### 3.2. Dense Multiscale Region Proposal Module

This module shares a similar structure with the SSD region proposal module. The SSD detection framework uses the VGG-16 Conv4-3 feature map as its first scale, which has a scale of a different feature compared to other layers. The remaining five scales of the SSDs framework are generated by applying convolution operations on VGG-16 Conv5-3 feature maps. Our experiment found that the feature detail of a small-scale object does not propagate through all hierarchical scales with such a framework.

**Figure 2:**
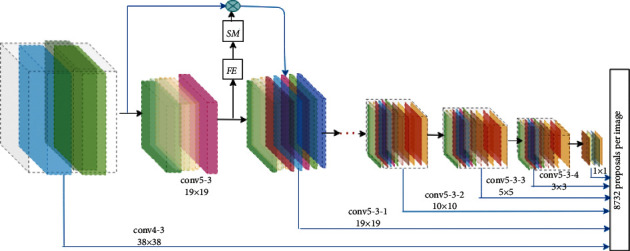
Our proposed region proposal module, here VGG-16 Conv5-3 layer feature map pass-through attention score feature extractor module FE, which is learned by 1 × 1 kernel. The output from FE pass-through the soft-max normalization SM to ensure a non-negative feature selector. Finally, the output of SM is multiplied with a low-layer feature map which enables a significant feature from the low-layer feature maps to pass through the higher layer, i.e., can avoid background jumble and semantic ambiguity.

The vanishing of the feature detail results in poor performance causing hurting bounding box learning, and the bounding boxes proposal layer has a high miss rate. Hence, to tackle this limitation we apply a focused attention selection mechanism [64, 65] on the VGG-16 Conv4-3 layer to blend with the Conv5-3 feature, that preserves a long-term dependence between layers. It results in the propagation of more detailed information about an object to a layer that contains semantic information while avoiding background clutter and semantic ambiguity. All hierarchies of the feature layer receive feature maps of their previous layer which makes an object's feature pass-through all layers.  Figure 2 shows how a lower-layer feature maps integrated with a higher layer feature. In this combination, a feature map of a Conv5-3 pass-through attention score (FE) is learned by 1 × 1 kernel as follows:

(1) $$\begin{array}{c} FE=W_{g}*Y\text{,} \end{array}$$

where *∗* denotes a convolution operation, *W*_*g*_ is the learned weight matrices implemented as a 1 × 1 convolution, and *Y* is the feature map of conv5-3. Then, the soft-max normalization is applied on a value of *FE* to get the feature selector, which assures non-negative selection of feature map.

(2) $$\begin{array}{c} SM_{i,j}=\frac{\exp^{FE_{i,j}}}{\sum_{i}^{W}\sum_{j}^{H}\exp^{FE_{i,j}}}\text{,} \end{array}$$

where, SM ∈ *R*^*W*×*H*×1^ and *S*_*i*,*j*_ are the score at position (*i*, *j*). A new lower-layer features is given by *X*_*i*,*j*,*c*_^*s*^=*SM*_*i*,*j*_*∗X*_*i*,*j*,*c*_. This operation gives a more relevant lower-layer feature. Here, *X*_*i*,*j*,*c*_ denote the value in *X* with a spatial location (*i*, *j*) at channel *c*.

There are six hierarchical convolution layers in the region proposal module. To improve layer feature map resolution and enable minor object features to reach the end of the feature proposal region, we combine feature maps from higher layers with feature maps from lower layers so that they can be detected. Background features propagate forward due to direct integration of layer's feature maps, reducing detection accuracy and increasing miss rate. As a result, we only use the feature selector to forward important feature maps from the lower layer to the higher layer.

This feature selector assigns attention scores to each local position on the low-layer feature map, indicating the low-layer features' importance. The attention score selector is learned by 1 × 1 × *c* kernel size, where *c* is the feature map depth. As shown in Figure 1, the first and last layer of the region proposal module is directly connected. However, the middle layers feature map is fussed, so based on this configuration the number of parameters is as follows: 1 × 1 × 512 kernel for the second layer and the remaining three layers each has 1 × 1 × 256 kernel size. Hence the total number of parameters is 1280.

The SSD's bounding boxes' width and height are defined by the aspect ratios (1, 0.5, 2, 0.333, and 3) with scales starting at 0.2 and growing linearly to the rightmost layer at a scale of 0.9. However, this design distribution does not best fit a common pedestrian dataset [5] because the equation [40] of the SSD's bounding boxes definition yields 50% of boxes with a width size greater than height resulting in misalignment between anchors and ground-truth bounding box features. As a result, we define bounding boxes shape as illustrated in the following equation:

(3) $$\begin{array}{c} Height=scale\times aspect\_ratio\times feature\_map\_height\text{,}\\ Width=scale\times aspect\_ratio\times feature\_map\_width\text{.} \end{array}$$

There is no standard for defining the number of model anchors and their characteristics. Nevertheless, a good decision is based on several factors. The problem type (the density of the objects in the dataset) i.e., are the objects in the dataset sparse or dense? Or if the objects in the image are large, small, or mixed and the smallest and largest boxes to be detected in the dataset are common considerations. Moreover, CNN's key characteristics of multiscale, regular deformation, and sparseness make it the optimal view, particularly for our problem of dealing with the dynamically changing nature of crowd density and the scale of various objects.

First, the bottom layers of CNN aggregate very fine, small-scale information, which is close neighbor information, and then, through the cascading of convolutional and subsampling operations, gradually aggregate information on a larger scale. Consequently, our anchor number and aspect ratio configuration are assigned based on these and the preceding factors. There are so many anchors for the lower layer because this layer's feature contains many objects, and as a layer's height increases, we reduce the number of anchors, because the feature may contain a few objects with large dimensions. Consequently, given the preceding factors, the configuration of the aspect ratio (1, 0.5, 3, 4, and 2) and the number of anchors (6, 6, 4, 4, and 4) at each layer of a region proposal yielded a satisfactory outcome after several experiments.

### 3.3. Adaptive Bounding Box Suppression Module

To demonstrate the effect of nonmaximum suppression (NMS) on occluded pedestrian detection, we examine various NMS techniques, such as greedy-NMS [66], soft-NMS [67], and our proposed modified soft-NMS called score-soft NMS. A greedy-NMS applies a hard threshold when deciding what should be retained or eliminated from K's bounding box neighborhood. Setting maximum and minimum threshold values has a cost, for example, suppressing all nearby detection boxes with a low threshold value increases the miss rate.

**Figure 3:**
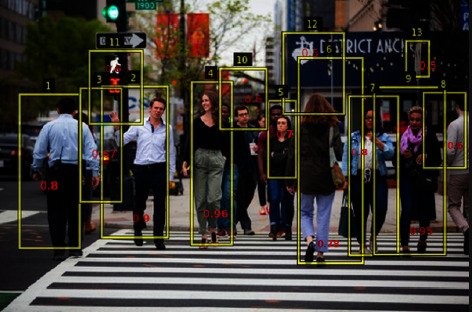
Our figure shows a pedestrian's detection score; with the help of the declining score function, occluded pedestrians can be detected with a low score value. When greedy-NMS is used, a pedestrian with label 9 overlaps with labels 7 and 8, results in a 0 score for both. The penalization of the score, on the other hand, aids in the detection of the occluded pedestrian.

Furthermore, using a high threshold value would result in more false positives, lowering average precision.  Figure 3 depicts the effect of greedy-NMS on a crowded pedestrian. A pedestrian with a label of 2 has neighbors labeled with 3 and 11; their score is 0.67 and 0.6, respectively. Because both boxes overlap significantly with label 2, greedy-NMS suppresses them and assigns a score of zero. While Soft-NMS decays the scores of 2's neighborhood, the score for detection boxes with a higher overlap with 2 should be decayed more, as they are more likely to be false positives. The time complexity of this algorithm is equivalent to that of soft-NMS, but the decaying function is different where *f*(iou(*M*, *b*_*i*_), di ff(*s*_*m*_, *s*_*i*_)) is the weighting function based on overlap. In each iteration of removing an anchor has a computational complexity of *O*(*N*), where *N* is the number of detection boxes.

Soft-NMS outperforms greedy NMS in terms of precision, especially when dealing with crowded pedestrians. However, the rescoring function based on the overlap parameter results in a low decay rate for a proposal having a small score value. Hence, to tackle this, we modified the soft-NMS of rescoring function by considering a proposal's score and overlap. For example, detection boxes with higher overlaps with 2's and a low objectness score decay more than the exact overlaps but with a high objectness score.

As shown in equation (4), decaying is an exponential function of the difference between a proposal having a high score and its neighbor's scores with their overlap value. This results in a detection box having a higher overlap and a lower objectness score with the highest score proposal decaying faster than a high score value. This update rule is applied every cycle, and the scores of all remaining detection boxes are updated. The algorithm's top, middle, and bottom boxes represent greedy-NMS, soft-NMS, and modified soft-NMS, respectively.

(4) $$\begin{array}{c} s_{i}=e^{-\left(di\;ff\left(s_{m},s_{i}\right)\right)}s_{i}e^{-\left(iou\left(M,b_{i}\right)\right)}\text{,} \end{array}$$

where ∀*b*_*i*_ ∉ *D*, *s*_*i*_ is the revised abjectness score of a proposal. The overlap-NMS technique is depicted in full in the algorithm, which includes the overlap and abjectness score-based weighting function.

## 4. Experiments and Results

### 4.1. Dataset and Evaluation Metrics

To evaluate the usefulness of the proposed approach, we used the Caltech dataset [5], which is a benchmark for testing pedestrian detection algorithms. The dataset is significant and difficult to evaluate, comprising around 10 hours of videos (at 30 frames per second) taken from a vehicle traveling through metropolitan traffic. In every frame of the raw Caltech dataset, the bounding boxes of pedestrian instances have been heavily annotated. In 250,000 frames, 350,000 bounding boxes with about 2,300 distinct pedestrians are classified. We employ the log-average miss rate in log-space in the range [0.02 to 1] to describe detector performance. Pedestrian height and proportion of occlusion have been used to construct various assessment settings. The following are the parameters utilized in this paper.

#### 4.1.1. Setups Involving Scale

Near (80 pixels or more), medium (30 pixels to 80 pixels), and far (30 pixels or less) are the three categories of pedestrian distribution in the dataset; the medium size is excellent for automobile systems. It is the most often used evaluation setting, and practically all pedestrian detection research studies use it as a standard evaluation benchmark. In this study, it is also the default setting for evaluation.

#### 4.1.2. Setups Involving Occlusion

The partial and heavy occlusion categories apply to pedestrians between 30 pixels to 80 pixels and have 1–35 and 36–80 of their body parts occluded, respectively. We test our methods on the updated annotations supplied by [18], which amend the mistakes in the original annotations and the Caltech dataset with the original annotations.

#### 4.1.3. Log-Average Miss Rate (LAMR)

Used to measure the performance of the object detector by comparing the log-average miss rate of the detection results compared to ground-truth data.

(5) $$\begin{array}{c} LAMR=\exp\;\left(\frac{1}{p}\underset{f}{\sum }\log\left(mr\left({argmax}_{fppi\left(c\right)\leq f}fppi\left(c\right)\right)\right)\right)\text{,} \end{array}$$

where, *fppi* is the number of false positives per image, *p* is the number of *fppi* reference points, and *c* is a confidence threshold *c* which is used as a control variable. By decreasing *c*, more detections are taken into account for evaluation resulting in more possible true or false positives, and possibly fewer false negatives, and *mr* is the miss rate which is equal to the division of the number of false negatives *fn*(*c*) by the sum of the number of true positives (*tp*(*c*)) and the number of false positives (*fp*(*c*)). For each *fppi* reference point the corresponding *mr* value is used. In the absence of a miss-rate value for a given *f* the highest existent *fppi* value is used as a new reference point.

### 4.2. Ablation Study

First, we conduct ablation experiments to evaluate the single-scale kernel properties based on the features produced from the VGG-16 base framework. We use recall rates at various IoU thresholds for evaluation. The results of the experiment are shown in Table 1. The Conv5-3-4 feature has the highest MR of 13. The impact of low-resolution features on proposal quality can also be noticed. MR increases as we progress from low-level to high-level features. Conv4-3 begins to decline, which may be attributed to a diminished depiction of the shallower layers. We contend that the low-resolution features are the reason for the poor performance.

To demonstrate this, we employ our modified VGG-16, multiscale feature extraction-based network (on the same set of region proposals). Table 1 of column 6 shows the MR result after modification of the VGG-16 base network. From the result, we can see that the MR of each layer of feature maps is enhanced. This fact reveals that multiscale feature extraction is vital for an effective proposal because it is large enough to capture the prominent characteristics of an image, allowing for a maximum effective receptive field with a shallow layer and sufficient signal reach at the base network's end layer.

### 4.3. Evaluation of the Dense Multiscale Region Proposal Detector

This section is devoted to exploring the effectiveness of the proposed dense multiscale region proposal detector. The feature maps from Conv4-3 with a resolution 38 × 38 of VGG-16 are our framework's first layer because they achieve the best performance among the different layers [40]. Then, using a focused attention selection mechanism [68], we blend the higher layer with the lower-layer feature to generate the remaining five hierarchical feature maps.

Table 2 shows the results of our proposed framework for all combinations of feature maps. The results show that the combination of features outperforms each CNN feature alone, such as SSD feature representation. The combination of Conv-4-3 and Conv 5–3 layer of VGG-16 achieves an MR of 9.1, which is slightly better than the best MR of 9.6 achieved in [34]. In their work, the best result is achieved by the combination of conv3-3 and the trous version of Conv4-3 takes extra computation time to recompute the conv4-3 features maps with the trous trick and adds background clutter and semantic ambiguity to a higher layer. While attention selection feature integration requires more computation time, it allows lower-layer feature maps to propagate forward if they have a high correlation with higher-layer feature maps.

As a result, more detailed information about an object is flowed to a semantic layer, avoiding background clutter and semantic ambiguity. This suggests that optimizing the effective receptive field of a base network and using an efficient feature integration algorithm can provide quality object proposals and improve detection accuracy.

### 4.4. Evaluation with respect to Occlusion

To demonstrate the importance of score-aware soft-max suppression in occlusion-aware pedestrian detection, we substitute Base − Mo de l+Region − Proposal of SSD greedy-NMS with soft-NMS and our suggested NMS. We investigate the applicability of our technique in various occlusion evaluation circumstances (none, partial, and heavy).  Table 3 shows the outcomes. This comparison is carried out using the MR metric, applied to all feature map hierarchies of the region proposal module. According to this analysis, the considerable improvement of nonmaximum bounding box suppression is crucial to enhancing the accuracy of occluded detection. It demonstrates one approach to dealing with occlusion aware-detection systems.

### 4.5. Comparison with State-of-the-Art Methods on Caltech with the Original and New Annotations

Table 4 compares our best results of dense multiscale feature extraction with score-soft pedestrian detection (DMSSPD) to state-of-the-art methods on the Caltech dataset with original and latest annotations. On the two benchmarks, our technique produced MR of 9.1% and 6.08%, respectively. Several research classes have produced better results than ours, but they all employ more complex structures and need higher processing costs.

For instance, the work [69] achieved an outstanding result. A single-shot convolutional multi-box detector for pedestrian candidates a classification system employing the concept of ensemble learning to improve the detection accuracy and classify the generated candidates, a novel soft-rejection fusion method to assign floating point labels to the generated pedestrian candidates, and a deep context aggregation semantic segmentation network that provides the pixel-level classification of the scene.

The aforementioned components make the model more computationally complex than our proposed method because our proposed method has three moving parts: a single-shot convolutional multi-box detector for pedestrian candidates, which is identical to [69]; a focused attention selection module with a complexity of 11c kernel size where *c* is the depth of six layers for propagating essential features to the next layer; and an adaptive bounding box suppression module is the weighting function. The computational complexity of each step is *O*(*N*), where *N* is the number of detection boxes. Furthermore, [72] utilized a multitask infusion framework for pedestrian detection and semantic segmentation joint subprediction.

The segmentation infusion layer yields more refined shared feature maps, which tend to illuminate pedestrians and facilitate pedestrian detection in a subsequent step. It consists of four elements, including weak segmentation supervision, proposal padding, cost-sensitive weighting, and stricter oversight. Because each pixel in the image is computed to identify a foreground feature, each component has numerous parameters and computational complexity.

## 5. Conclusion

The detection of pedestrians is a primary concern. In ideal settings, such as a pedestrian with a comparable scale (above 80 pixels in height) and complete visibility, the current state-of-the-art produces an excellent outcome. Despite significant advancements, recognizing small-scale pedestrians remains a source of the problem. As a result, to address this issue, we present an average model weight ensemble framework that can learn a variety of mapping functions and allows features to have an equal impact within the receptive field by spreading the concentration of a sensory field center to the periphery. We also improve an adaptive bounding box suppression that maps objects to nearby points via dimensionality reduction.

## Figures and Tables

**Algorithm 1:**
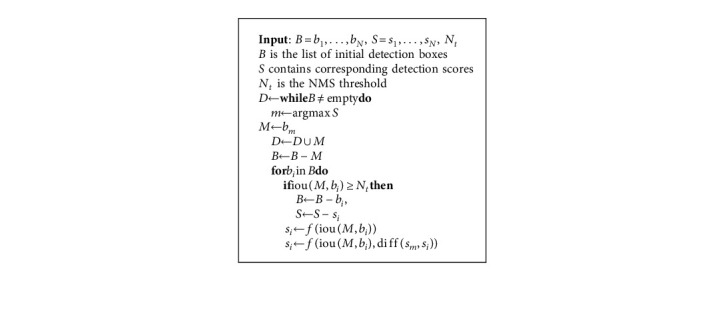
The algorithm shows the modified version of Soft-NMS. The last box explicitly shows our proposed objectness score decaying function, a function of IOU and score parameter. The function revise the detection scores by scaling them as Gaussian function of overlap and objectness score.

**Table 1:** Comparison between the base network VGG-16 and our proposed modified base module for a small-scale pedestrian on each hierarchy of layers.

Feature map	Resolution	Base network	MR	Base-network	MR
Conv4-3	38 × 38	VGG-16	9.6	Modified VGG-16	9.1

Conv5-3	19 × 19	VGG-16	10.2	Modified VGG-16	10

Conv5-3-1	10 × 10	VGG-16	10.8	Modified VGG-16	10.3

Conv5-3-2	5 × 5	VGG-16	11.5	Modified VGG-16	11.5

Conv5-3-3	3 × 3	VGG-16	12.8	Modified VGG-16	12.3

Conv5-3-4	1 × 1	VGG-16	13	Modified VGG-16	12.6

**Table 2:** The detection performance (MR) of the region proposal module layers for medium and small-scale pedestrians on average.

SSD feature map	Base network	Resolution	MR	Modified SSD feature map	MR
Conv4-3	VGG-16	38 × 38	11.5	Conv4-3	11.10
Conv5-3	VGG-16	19 × 19	12.32	*A* ← *conv*4 − 3, *conv*5 − 3	10.8
Conv5-3-1	VGG-16	10 × 10	12.65	*B* ← *conv*5 − 3 − 1, *A*	10.9
Conv5-3-2	VGG-16	5 × 5	13.02	*C* ← *conv*5 − 3 − 2, *B*	12.1
Conv5-3-3	VGG-16	3 × 3	14.36	*D* ← *conv*5 − 3 − 3, *C*	13.01
Conv5-3-4	VGG-16	1 × 1	16.2	Conv5-3-4, D	13.98

**Table 3:** The effect of nonmaximum suppression under different occlusion settings and detection performance (MR) under different scales.

NMS	Detection performance (MR) under different scales and occlusion									
	VGG-16 + RPN					Proposed method				
	Nea	Med.	Part	Hea	NoN	Nea	Med	Part	Hea	NoN
G-NMS	58.09	2.59	78.77	27.64	11.33	58.09	2.23	77.77	78.8	27.64
Soft-NMS	56.31	2.59	76.62	26.54	9.95	56.31	2.32	72.62	24.54	9.11
Score-soft-NMS	54.35	1.66	74.72	20.4	8.15	54.35	1.12	71.03	22.8	8.23

**Table 4:** Comparison of our works with other works on Caltech with the original (MR^*O*^).

Methods	MR^*O*^	MR^*N*^
F-DNN [69]	8.65	—
HCD [34]	9.53	6.41
PDM [70]	10.2	—
Hyper learner [71]	—	5.5
SDS-RCNN [72]	7.36	6.44
CompACT-deep [73]	11.75	9.15
MCF [74]	10.40	7.98
TLL-TFA [75]	7.39	—
DMSSPD	9.1	6.08

## Data Availability

The dataset is publicly available. We cite the dataset in Section 4 for anyone interested in using it.
